# Delivering Inclusive Cultural Offers for Social Prescribing: A Realist Evaluation Involving Older People From Global Majority Backgrounds and Cultural Sector Providers in the UK

**DOI:** 10.1111/hex.70550

**Published:** 2026-02-14

**Authors:** Debra Westlake, Kamal R. Mahtani, Geoffrey Wong, Jordan Gorenberg, Marta Santillo, Kerryn Husk, Sofia Vougioukalou, Ruthanne Baxter, Shoba Dawson, Caroline M. Potter, Harriet Warburton, Beth McDougall, Stephanie Tierney

**Affiliations:** ^1^ Nuffield Department of Primary Care Health Sciences University of Oxford Oxford UK; ^2^ Faculty of Health, University of Plymouth Plymouth UK; ^3^ Centre for Adult Social Care Research & Clinical Trials Unit, Cardiff University Cardiff UK; ^4^ Library and University Collections, University of Edinburgh Edinburgh UK; ^5^ Sheffield Centre for Health and Related Research, University of Sheffield Sheffield UK; ^6^ Gardens, Libraries and Museums, University of Oxford Oxford UK

**Keywords:** cultural sector, engagement, global majority, minority ethnic, older people, realist, social prescribing

## Abstract

**Introduction:**

Research shows cultural activities benefit older people's wellbeing, but little is known about why individuals from global majority (minority ethnic) groups engage less with the mainstream cultural sector, or how it could adapt to meet their needs and encourage engagement. The TOUS study (Tailoring cultural Offers with and for diverse older Users of Social prescribing) investigated the question: W*hat tailoring is needed, how, when and for whom, to optimise cultural offers as part of social prescribing for older people (aged 60*+*) from global majority groups?*

**Methods:**

The TOUS study involved focused ethnographies with six cultural‐sector organisations throughout the United Kingdom and 11 key informant interviews with cultural providers. A realist approach was adopted, resulting in the development of a programme theory.

**Results:**

The programme theory has three pillars: (1) broker, hook, and opportunity, which support entry into cultural spaces to provide immediate benefits; (2) safety and trust, power‐sharing, and meaning, which sustain engagement; and (3) transformative outcomes, including lasting relationships, confidence, and exploring identities.

**Conclusions:**

With intentional engagement and relational practices, organisations can foster inclusive cultural participation and support well‐being in later life.

**Patient or Public Contribution:**

The TOUS study was guided by a public involvement group of six contributors (aged 60+ from global majority/minority ethnic backgrounds) who shared experiences of creative practice, and advised on data collection, analysis, model development, and dissemination. The study also involved collaborative analysis of data at case sites.

## 1 Introduction

Evidence suggests that engaging with the creative and cultural sector improves health and well‐being [1, 2]. However, social, economic, geographical and cultural factors can shape access to creative spaces [3]. Older adults are a heterogeneous group influenced by these factors as well as by the impacts of ageing [4]. Nevertheless, when cultural activities are intentionally designed to meet their needs, participation can deliver significant health and well‐being benefits to older people, such as cognitive stimulation, social connections and improved mood [1, 5, 6, 7, 8, 9, 10, 11].

Social prescribing is one path through which older people may be connected to cultural provision. It involves supporting people with social issues affecting their health and well‐being (e.g., impacts of long‐term conditions, loneliness, worries about money, housing problems). In England, link workers are employed (mainly via a primary care or voluntary‐community organisation) to deliver social prescribing. They talk with people about their lives and, where appropriate, connect them to local support, often via community or voluntary groups.

Research indicates that referrals to social prescribing are lower among people from global majority groups [12, 13, 14] and that global majority communities access mainstream cultural offers less than white British populations [15, 16, 17], despite attempts to increase diversity, for example by decolonisation processes in museums [18].

In this paper, we use the term ‘global majority’ rather than ‘ethnic minority’ or ‘minority ethnic’ groups. Recognising that terminology is contested and also situational, ‘global majority’ is used as it is a preferred term by United Kingdom (UK) cultural sector organisations who are the focus of this project. It is also adopted by bodies such as the National Council for Voluntary Organisations [19].

A scoping review we conducted found few studies explaining why older people from global majority communities face barriers to creative and cultural activities [20]. Available research focused on challenges experienced by individuals: language barriers, lack of confidence, difficulty accessing transport, unfamiliarity with the cultural setting and/or activities, and older people having low energy. The review found few studies or recommendations to guide organisations in engaging older people from global majority communities, leaving providers with little direction on how to design accessible activities [20]. Link workers may not refer to provision in cultural settings due to limited awareness of such offers and their benefits [21]. Our previous research also found that link workers' assumptions about individuals' interest in cultural activities influenced their recommendations [5].

In an earlier study, we developed a programme theory that highlighted four pathways through which older people's health and well‐being could be improved by engaging with cultural provision: *immersion* (offering temporary escape); *psychological holding* (fostering safety and belonging); *connection* (enabling relationships and interaction); and *transformation* (promoting growth through learning and confidence) [5]. This study did not examine whether ethnically diverse older people have different needs, or how cultural providers tailor offers to encourage diversity. Stakeholders highlighted this as a priority for future research, leading to the TOUS study (Tailoring cultural Offers with and for diverse older Users of Social prescribing). The TOUS study investigated how cultural spaces, groups or activities, which might form part of a social prescription, can be adapted by cultural providers to enhance engagement among older adults (aged 60+) from global majority communities.

## 2 Methods

The TOUS study addressed the question: *What tailoring is needed, how, when and for whom, to optimise cultural offers as part of social prescribing for older people (aged 60*+*) from global majority groups?* It aimed to develop evidence‐based recommendations about producing accessible, acceptable and appropriate cultural offers to support the well‐being of older people from diverse backgrounds. The study methodology was designed to test (confirm, refute or refine) the programme theory from our previous study on tailoring cultural offers for older people [5].

### 2.1 Realist Research

Realist evaluation is a theory‐driven approach that identifies causal factors by iteratively developing and testing a programme theory [22]. This involves mapping assumptions about how an intervention works and constructing causal explanations in the form of context–mechanism–outcome configurations (CMOCs) to explain what works, for whom, in what circumstances, and why. Key realist terms are defined in Table 1.

**Table 1:** Realist terminology definitions.

Realist term	Definition
Context	The conditions, circumstances or settings in which an intervention takes place and which cause mechanisms to occur. This includes structural factors such as social, economic, political and organisational aspects, but also individual factors such as cultural and social norms and relationships. In the TOUS study, the context of the prior experiences of the older person and their potential broker, as well as aspects of the cultural sector organisation are salient (see table of CMOCs).
Mechanism	The underlying processes—comprising resources provided by the intervention and the reasoning or responses of participants—that explain how and why certain outcomes are generated. Mechanisms include responses that individuals may or may not be aware of. In the TOUS study, mechanisms such as development of trust or feeling safe are important for engagement in the cultural sector.
Outcome	Mechanisms alter the behaviour of participants; outcomes are the effects or results that follow when mechanisms are triggered in a given context. Outcomes can be intended or unintended, positive or negative. leading to different outcomes within certain contexts. In the TOUS study, outcomes such as increased engagement and sustained attendance were explained by specific contexts that activated mechanisms enabling these results to occur.
Context‐mechanism‐outcome configuration CMOCUK	These heuristics are used to show patterns in the relationship between context, mechanism, and outcome that explain how particular results arise in specific circumstances. These are sometimes also called micro‐theories.

### 2.2 Defining Cultural

The TOUS study incorporated definitions of ‘cultural’ from different sources [2] including data collected for our previous study [23]. We considered the following broad areas as cultural offers in the TOUS study: (a) heritage (e.g., museums, historical venues, curated green spaces); (b) performance (e.g., music, theatre, dance); (c) visual arts (e.g., photography, crafts, painting); (d) books/literature (e.g., poetry, libraries); (e) audio‐visual (e.g., films). Exploratory conversations with organisations offering activities to our target population, during early stages of the TOUS project, led us to expand our definition to include (f) cultural and religious events such as festivals that might include a range of elements such as dancing, singing and food [24].

### 2.3 Data Collected for the TOUS Study

A study protocol was published on the study webpage before commencing data collection [25]. The initial phase of the TOUS study comprised a scoping review of the existing literature [8] and a mapping exercise, which involved an online questionnaire targeted at cultural sector providers [24]. The purpose of the questionnaire was to identify the nature and geographical distribution of current cultural provision for older adults from global majority backgrounds across the UK. It also sought to gather insights into how cultural organisations adapt or tailor their programmes to better meet the needs of these communities.

Questionnaire responses and follow‐up conversations with respondents informed the identification and selection of six provider organisations for focused ethnographies (our case sites) [26, 27]. Sampling aimed for variation in geography, types of cultural venue and kinds of activity offered. Case site organisations offered varied creative activities (see Table 2).

**Table 2:** Cultural/creative activities offered at each case study site.

Site	Heritage	Performance	Visual arts (includes crafts)	Books/literature	Audiovisual	Food and festivals
Site 1	0	1	1	1	1	1
Site 2	1	1	1	0	0	1
Site 3	1	0	0	1	0	1
Site 4	1	1	1	0	1	1
Site 5	1	1	1	1	0	1
Site 6	0	1	1	1	1	1
Totals	4	5	5	4	3	6

Cultural venues (five from across England and one from Wales) included theatres, libraries, a museum, a day centre for older people and a women's centre.

Data collection for the focused ethnographies was undertaken between May 2024 and March 2025. It involved:

- Participant observation—researchers spent 1–5 days at an organisation, participating in and observing activities (e.g., knitting, singing, theatre) and making written notes (total 17 days).
- Interviews—15 interviews with cultural providers were conducted in‐person or via video calls. Interviews lasted from 24 to 49 min. They were audio‐recorded and transcribed.
- Storytelling—involved collecting, editing, and synthesising personal narratives given by those engaging in or providing cultural activities (defined as Storytellers) to understand the most significant changes they experienced as a consequence [28]. Storytellers were 16 older people from global majority backgrounds and 11 staff or volunteers from cultural organisations (*n* = 27 stories). Conversations lasted for 22–73 min and were recorded, transcribed, and condensed into two‐page summaries that preserved the Storyteller's voice and were approved by them. This approach surfaces underlying narratives and meaning—important for developing theory in this study. The Storytelling approach is described in more detail elsewhere [29].

We sought variation in the age, language, ethnicity, and role (e.g., provider or older person) of people involved in the research (see Tables 3 and 4).

**Table 3:** Roles, gender, age of people involved in case study sites data collection.

Role	*N*	Female	Male	Mean age	Age range
Participant (older person)	16	15	1	68	60–91
Managers	6	4	2	60	46–75
Producers	2	1	1	40	35–45
Artists/facilitators	5	3	2	48	29–60
Engagement/programme coordinators/community development workers	8	5	3	49	35–67
Link workers	2	2	0	47	42–52
Volunteers	1	1	0	63	N/A
Admin and outreach	2	2	0	38	30–47
Totals	42	33	9		

**Table 4:** Ethnicity of people involved in case study data collection.

Ethnicity (self assigned)	Participants (older people)	Managers	Other workers	Link workers
Black British	4	1	3	1
Black African	2	0	0	0
Chinese	3	0	1	0
Pakistani/Welsh Pakistani	3	1	2	0
Indian/British Indian	1	0	1	1
African Caribbean	1	0	0	0
British Iraqi	1	0	0	0
British Bangladeshi	0	1	1	0
British Asian	0	0	1	0
Lebanese	0	1	0	0
Irish/African	1	0	0	0
Arab Jordan	0	0	1	0
African/Indian British	0	0	1	0
White British	0	2	7	0
Totals	16	6	18	2

In addition to people involved in the focused ethnographies at case study sites (listed above), a subset of questionnaire respondents (*n* = 11) participated in key informant interviews (42–65 min in length). They were providers from organisations not specifically engaging with older people from global majority communities who gave insights into the challenges and opportunities they had experienced when trying to do so. These interviews were conducted between March and June 2024. Their job roles were related to learning and engagement (*n* = 5), being an artist or facilitator (*n* = 3), or well‐being manager/officer (*n* = 3). Tables 5 and 6 provide more details.

**Table 5:** Key informant interviewee characteristics: Gender and age.

Key informant interviews^a^	*N*	Female	Male	Mean age	Age range
Interviewees completing demographic information	10	8	2	49	34–69

^a^ One interviewee did not provide these details.

**Table 6:** Key informant interviewee characteristics: Ethnicity.

Key informant interviews: Ethnicity (self assigned)	*N*
European	1
Polish	1
White British/Jewish	1
Indian	1
British	2
White Welsh	1
White British	3
Not declared	1
Total	11

### 2.4 Analysis

Early analysis was informed by the programme theory from our previous project [5]. During the analysis process, we held meetings with research team members, public contributors, and an advisory group to explore ideas from the data and to draw draft conceptual maps. We developed a framework to thematically code data from interviews, observations and stories using QSR NVivo 15. We made notes about where these codes might be developed into realist CMOCs.

As the study progressed, analysis sessions were held at case study sites involved in Storytelling; details of these sessions are reported elsewhere [30, 31, 32]. Each discussion session (*n* = 3) was attended by between 15 and 20 participants including Storytellers, older people from global majority groups, cultural sector workers and other stakeholders. Storytelling is a collaborative method ensuring multiple voices are included in analysis. Storytellers and stakeholders read stories in advance and reflected on key themes during facilitated discussions. Researchers incorporated these themes into the analysis.

As part of the analysis, we considered whether CMOCs from the previous study [5] were relevant and whether modifications were required to apply them to data collected in the TOUS study. We added to these CMOCs, writing new ones derived from our TOUS study dataset. The final TOUS model was refined through team meetings, cultural sector stakeholder input, and discussions with the study's public involvement group [33].

### 2.5 Public Involvement

The study public involvement group consisted of six contributors aged 60+ years who identified as part of the global majority, lived in England or Wales, and actively participated in creative activities. They advised on data collection, giving feedback on interviewing techniques, data analysis, theoretical model development and dissemination. A flexible involvement method was employed in which group members stated their preferred engagement style (i.e., online, face‐to‐face, one‐to‐one and/or group meetings) and contribution methods (i.e., question and answer elicitation, creative methods such as collaging and photo elicitation, and ranking/rating activities).

## 3 Findings

### 3.1 The Programme Theory

Elements of our previous study's programme theory remained applicable [5]. However, TOUS study data, gathered with global majority groups, highlighted distinct contexts requiring alternative frameworks. The TOUS programme theory is organised around three pillars of a cultural journey (Entry, Engagement, Benefits). Underpinning these pillars are the characteristics and experiences of the person. Each pillar is supported by realist CMOCs (see Tables 7, 8, 9; see also Supporting Information S1: File S1 for supporting quotes from data). For brevity, below we use the term ‘older people’ to mean ‘older people from global majority communities.’

**Table 7:** CMOCs and summary statement relating to Pillar 1: The concepts of ‘broker’, ‘hook’ and ‘opportunity’.

No.	CMOCs—Broker
1	When a broker is trusted and empathetic [C] their suggestions are seen as credible [M] so they are listened to and acted upon [O]
2	The broker provides information and reassurance [C] reducing an older person's fear [M] so they feel safe entering a cultural venue [O]
3	When they have insider experience of an activity [C], the broker can talk about it with conviction [M] providing a positive message to older people about the benefits to expect [O]

No.	CMOCs—Hook
4	When a choice of activities is offered, and includes something familiar to the older person [C], they feel comfortable and confident with what is involved [M] so are attracted to take part [O]
5	When the benefits of activities are made clear to the older person [C] and this resonates with what they need at that time (which might include a challenge) [M], this prompts them to want to get involved [O]
6	Seeing peers engaging in and enjoying cultural activities [C] sparks an older person's interest [M] making what is offered attractive to them [O]

No.	CMOCs—Opportunity
7	If a change in circumstances removes constraints or barriers experienced by a person [C] it gives them a sense of freedom [M] meaning they are more likely to engage with cultural offers [O]
8	When people realise a change is needed in their life [C], they become more receptive to what is available [M] and capitalise on opportunities for creative expression [O]
9	When barriers to access are removed [C], it feels feasible, convenient or low risk [M] so people are more likely to want to engage with cultural offers [O]

*Note:* **Summary statement for Pillar 1:** When a trusted person (broker) presents a cultural offer in an engaging way (hook), at a moment when the individual is open to it (opportunity), older adults from global majority groups are more likely to take the first step into a cultural space or activity.

**Table 8:** CMOCs and summary statement relating to Pillar 2: The concepts of ‘safety and trust’, ‘power sharing’ and ‘meaning’.

No.	CMOCs—safety and trust
10	When cultural institutions provide a welcoming and non‐judgmental environment [C] participants develop trust in the space and facilitators [M] leading to increased engagement [O]
11	When facilitators build trusting relationships with people over time [C] participants feel valued, safe and comfortable returning [M] resulting in sustained attendance [O]
12	When participants become familiar with a cultural setting and people there over repeated visits [C], they become less anxious about attending [M] enabling them to feel they belong [O]
13	When cultural organisations make it clear they will enact diversity and inclusion practices [C] participants develop trust in the space and facilitators [M] and are more likely to take part [O]
14	When participants see that cultural spaces do not represent them [C] they do not attend [O], believing they are not welcome [M].

No.	CMOCs—power sharing
15	When cultural activities are co‐created with participants [C], they feel stronger personal investment [M] leading to longer‐term engagement [O]
16	When cultural organisations facilitate and encourage power sharing [C] it is more meaningful to participants [M] so they become active contributors (rather than passive recipients) [O]

No.	CMOCs—meaning
17	When cultural activities resonate with participants’ personal histories and identities [C], this fosters a sense of representation and inclusion [M] meaning that older people feel a stronger connection to the organisation [O]
18	When participants feel that cultural activities add structure and routine to their day [C], they have something to look forward to [M] which leads to personal fulfilment and a sense of purpose [O]
19	Organisations (facilitators, artists and other staff) that understand older people's socioeconomic, cultural, language and religious reference points (the whole person) are flexible and adapt what they offer in response [C], which makes individuals feel understood and valued [M] so they are more willing to keep participating [O]

*Note:* **Summary statement for Pillar 2:** When cultural spaces work with participants to create a welcoming environment of safety and trust, participants feel a sense of belonging and ownership over the activities, leading to deeper engagement, continued participation, and meaningful connections with others.

**Table 9:** CMOCs and summary statement relating to Pillar 3: Benefits of activities.

No.	CMOCs—immediate benefits (* denotes CMOCs from previous study [5])
20	*When an older person finds the cultural offer stimulating (C), they experience a short‐term escape from their problems (O) because they enjoy and are absorbed by the activity (M)
21	Trying new creative activities outside of usual experiences [C] gives participants a sense of accomplishment [M] which they find pleasurable and invigorating [O]
22	When participants enjoy the cultural activity [C], they feel a sense of pleasure [M] and are therefore more likely to continue with it [O]
23	When participants are in a safe space with permission to engage in activities (such as dance) [C] they can release inhibitions [O] because they feel they are not being judged (by certain societal or cultural norms) [M]
24	Taking part in cultural activities which promote the expression of emotions and experiences [C] leads to a sense of release [O] because participants can surface these feelings and start to process them [M]
25	* When the cultural offer provides a social component (C), older people feel less lonely (O) because they have been facilitated to engage in human interactions (M)

No.	CMOCs—intermediate benefits
26	When cultural sector organisations provide a safe space for participants to come together [C], they feel a sense of connection and belonging [M] so feel freer to explore their creativity [O]
27	When participants taking part in cultural activities are supported to share and process challenging experiences [C], they feel heard and validated [M] and therefore experience a stronger connection with other participants and facilitators [O]
28	People who have developed sustained relationships through the cultural activity [C] share experiences and difficult emotions more readily [O] because they have trust in these relationships [M]
29	Cultural sector activities that surface differences and similarities between multiple heritage groups [C], foster awareness about others’ traditions and languages [M], breaking down barriers between participants [O]

No.	CMOCs—longer‐term *personal* transformational benefits (* denotes CMOCs from previous study)
30	*When cultural offers enable participants to experience or learn new things (C), their self‐esteem and confidence increase (O) because they are encouraged to try things outside of their comfort zone (M)
31	When participants take part in cultural activities in an environment of trust [C] they may explore and try out new identities [O] because they are less fearful of negative judgement from others (M)
32	When the cultural sector gives people who have supressed (or not explored) their creative talents the opportunity and encouragement to do so [C], they feel emboldened [M] and can (re)discover their creativity [O]
33	Engagement in a cultural sector activity [C] can act as a springboard to other creative activities [O] because people have developed confidence in their abilities to try new things [M]

No.	CMOCs—longer‐term *societal* transformational benefits
34	When a cultural activity provides participants with the skills and opportunities to challenge bias and discrimination in wider society (C) they are more likely to do so (O) because they feel supported and emboldened (M)
35	When family participants see a relative taking part in cultural activities outside their comfort zone and experience [C], they feel proud (O) because their relative is achieving something unexpected [M]
36	When participants routinely and actively take part in structured activities outside the home [C], family participants feel less worried about them [O] because they can see their relative has a sense of purpose and is less isolated (they are living their lives well) [M]

*Note:* **Summary statement for Pillar 3**: When people have developed sustained relationships through engagement with the cultural sector, a process of transformation is possible fostered through personal growth, shifts in identity and healing of past experiences. Transformation can occur beyond the individual as these transformations are witnessed by family, community and wider society.

### 3.2 Underpinning Characteristics—How Life Stories and Experiences Shape Access to Cultural Activities

Older people's stories powerfully showed how past experiences of discrimination created anxiety about entering unfamiliar cultural spaces. Structural inequalities associated with intersectional factors (e.g., social class, ethnicity, religion, gender or migration status) led to many individuals feeling unwelcome in white, middle‐class cultural sector settings and organisations. Ageing processes, such as reduced mobility or cognitive capacity, together with economic insecurity, intensified exclusion by heightening barriers to cultural spaces, including travel distance, transport costs, entrance fees, and physical access. Where spoken or written English was the predominant language in a cultural setting, this could be a significant constraint. These factors meant that creative and cultural opportunities were often discounted by participants:

“I've always been interested in theatre, but I never ever had the courage to come and watch a play. Being mixed race and working class, I always felt it's not for me. There'll be nobody at the theatre that will represent me. So, I just kept away. With the [drama activity], I thought it'll just be all white people, staring at you thinking: what's she doing here? Then I spoke with a friend, and she said just give it a try, it's only an introduction.”

(Site 6_Participant 03).

Nevertheless, participants' stories showed they were resourceful, navigating challenges and experiences of exclusion. This meant they were able to engage in and benefit from cultural sector activities. To do so, they first had to enter the cultural space.

### 3.3 Pillar 1—Entering the Cultural Space

Older adults from global majority groups described stories of courage and resourcefulness as they stepped into cultural activities for the first time. Three elements helped them when entering new cultural spaces: a ‘broker’, ‘hook’, and ‘opportunity’.

#### 3.3.1 Broker

A ‘broker’ is someone who introduces cultural activities and creates a bridge to participation. The role of broker was not present in our previous study involving mainly white British older people [34] and appears to be particularly important for global majority participants.

Brokers were individuals who could fast‐track trust as they were regarded as credible, empathetic and aware of challenges experienced by older people from a global majority group. Often, they were individuals who had similar experiences, traditions and languages (e.g., a friend, neighbour). Brokers could also be community development workers or leaders from trusted organisations. In some cases, brokers were healthcare professionals (including link workers) or adult children who, concerned about a relative's isolation or inactivity, encouraged them to try local cultural opportunities.

The broker's suggestions were listened to and taken seriously. They could help break down negative preconceived ideas people had of a cultural setting and helped an older person to feel at ease, sometimes by accompanying them on a first visit.

The term ‘super‐connectors’ was used by one Storyteller to denote brokers who recommended cultural activities with conviction due to their insider knowledge, who provided first‐hand evidence of the cultural activity's benefits. It was noted that super‐connectors may require support from the organisation as they could feel disappointed when someone did not join a cultural group or activity, since opportunities would not suit everyone.

A repeated concept across the data was the importance of outreach. Partnering with local organisations was essential to gain the insight of older people from global majority communities into what cultural provision they valued and how it should be delivered. Time may be required by cultural organisations to develop such connections and to identify a receptive broker; our key informant interviews suggested that unsuccessful attempts at this may occur before finding an ideal person for this role.

#### 3.3.2 Hook

A ‘hook’ attracts someone to take part in a cultural offer or activity. It is what sparks their interest or curiosity; like the first line pulling a reader into a novel. In our data, a hook included a familiar and comfortable activity aligned with previous experiences (e.g., knitting or singing), something rooted in cultural references (e.g., African drumming, Bollywood music), or conversely, an opportunity to try something new and personally challenging (e.g., acting on stage for the first time). A broker was often able to identify a personalised hook that made the activity appealing to a potential participant.

#### 3.3.3 Opportunity

Data highlighted that the right moment, or ‘opportunity’, had to come along for an older person from a global majority group to engage in cultural activities. This concept reflected a sense of release from constraints, creating space for new perspectives, or experiences. This freedom may be:

- Attitudinal—having the ‘headspace’ to be ready to engage, such as after retirement or during life changes that spark a desire for creativity (e.g., post‐COVID).
- Relational—after a bereavement or when caregiving duties eased (e.g., due to grandchildren growing up).

Opportunities were also created by cultural sector organisations in material ways: holding activities in accessible venues, reachable by public transport, offering free parking and transport. Low or zero membership fees or funded places were also important, as well as considering timings of activities to allow for caring responsibilities such as picking up grandchildren from school. Crucially, the location needed to be perceived as inclusive (explored in Pillar 2) (see Table 7 for CMOCs relevant to Pillar 1).

### 3.4 Pillar 2—Engaging With the Cultural Offer

After the first step into a cultural space or activity, three mechanisms shaped how institutions sustained engagement with older adults from global majority backgrounds: ‘safety and trust’, ‘power‐sharing’, and ‘meaning’. These were within the organisation's control, delivered through staff, volunteers, artists, and the space itself.

#### 3.4.1 Safety and Trust

Safety and trust were fundamental to sustained engagement in cultural activities. If individuals did not feel safe in a space—whether physically, emotionally, or socially—they were unlikely to participate fully or return. Our data highlighted that safety was not just about the absence of harm, but also fostering an environment of psychological comfort, where individuals felt secure expressing themselves without fear of judgement or exclusion. Safety from discrimination was not merely stated as a policy but actively demonstrated through challenging inequities. At one site, a participant highlighted how staff reading out an Equity Safe Spaces Statement https://www.equity.org.uk/advice‐and‐support/dignity‐at‐work/creating‐safe‐spaces#safe‐spaces‐statement), at the start of sessions, made them confident to challenge discrimination.

Sense of safety was optimised through the smell of familiar food or recognisable sounds (e.g., music, language) within the cultural space. It might come from participants observing others who looked like them engaging in a cultural activity and seeing their enjoyment. It might come from artefacts (e.g., books, pictures) that reflected or resonated with a community. Safety was also supported by women‐only spaces for faith communities, where activities like singing, dancing or acting might be considered inappropriate in mixed‐gender groups.

Trust was built over time through repeated interactions with people and with the institution or setting itself. Facilitators (staff, volunteers and freelance artists) and peers played a key role in making individuals feel welcome, heard, valued, and respected. This was particularly important for older people from global majority backgrounds, who may have experienced exclusion or discrimination in other areas of life, making them guarded about new situations.

Smiling and friendly body language and gestures, such as offering food and drink, were powerful non‐verbal cues conveying reassurance, especially when verbal communication was limited due to language differences. When trust was established, participants became more confident taking part in activities and forming social connections.

#### 3.4.2 Power‐Sharing

This refers to the active involvement of older people from global majority backgrounds in shaping cultural activities. Successful case sites balanced structured and responsive cultural programming (often through co‐creation). When facilitators listened to and adapted to participants' preferences and needs, a reciprocal rather than transactional relationship was created. Older people saw themselves as active contributors rather than passive recipients and they felt invested in the activities, leading to stronger commitment. Belonging was crucial, as participation in the design of activities reinforced the idea that the space was ‘for them’.

In our data, power‐sharing took many forms: including participants influencing activity programming, shaping activities (e.g., formulating the script for a play, choosing the music or songs), or leading aspects of a cultural offer, perhaps by becoming a volunteer.

#### 3.4.3 Meaning

Cultural offers needed to be enjoyable while also resonating with lived experiences and identities to ensure meaningful, lasting participation. What felt significant and meaningful varied across individuals. Co‐creation, by centring participants' voices, made activities more relevant and responsive.

Meaningful engagement could involve cultural recognition—an older person seeing their heritage, traditions, or language reflected in activities. Culturally‐informed facilitators, who were often from similar communities to participants, adapted activities to respect religious and cultural beliefs. For example, in a South Asian Muslim community, women's participation in a festival performance was facilitated by organising gender‐segregated rehearsals and scheduling separate performance times, aligning with cultural norms of modesty and gender interaction. When institutions acknowledged and celebrated diverse histories and narratives, participants felt a stronger sense of inclusion and belonging (see Table 8 for CMOCs relevant to Pillar 2).

### 3.5 Pillar 3—Benefits of Being Involved in the Cultural Sector

#### 3.5.1 Immediate Benefits

In common with our previous study [5], the immediate sense of connection to a group of like‐minded individuals was compelling and restorative, particularly where people had previously felt excluded in cultural venues. It was an opportunity to release inhibitions in a safe space and to be heard and understood by others who shared similar cultural heritage and experiences. Participants also described a deep enjoyment—being absorbed in something creative gave them a break from daily worries and responsibilities. This offered respite, both emotionally (a mental escape) and practically (time away from caring or community duties). Enjoyment often came from a sense of achievement—like learning a new skill or overcoming insecurities about their creativity. For some, it reawakened a love for art or performance, put aside earlier in life due to work or family demands.

Meaningful engagement facilitated emotional release (catharsis). Responses were sometimes stirred by the activity itself—such as during a drama performance—or emerged indirectly, through the shared experience of creating something or celebrating together with others who had similar life journeys. This was powerful for people who had not previously explored feelings linked to being marginalised or overlooked.

#### 3.5.2 Intermediate Benefits

Some participants were content with short‐term benefits from occasional cultural involvement. However, longer term, sustained attendance was more likely to produce enduring benefits. For many participants, attending a weekly cultural activity provided stability and a sense of purpose. Having a regular event to look forward to was particularly beneficial for those experiencing isolation or lacking other social outlets. Regular engagement with creative work could foster meaningful social connections between members, volunteers and artists, particularly where the projects were collaborative, such as making a piece of artwork, planning a festival, or singing in a choir. The process of building skills and working together was, in most cases, as important as the final product or performance, enhancing a sense of belonging.

Sustained relationships continued outside cultural sessions, with group members showing caring and interest by checking in on each other or celebrating special occasions together. It was common across sites for participants to speak of the group as showing “love” and being “like a family”. This deep connection was particularly salient for displaced and migrant communities away from their own relatives. A virtuous cycle of sustained engagement and deepening relationships was a hallmark of these intermediate benefits; this could occur within a particular heritage or ethnic minority community, but also at some sites cross‐cultural connections were fostered, breaking down barriers as common experiences and emotions were shared via cultural activities.

#### 3.5.3 Longer Term—Transformational Benefits

Our previous study [5] highlighted the transformative power of engagement with the cultural sector for older people, contributing to increased confidence and self‐esteem. In the TOUS study, sustained, trusting relationships were key to unlocking the door to longer term transformational impacts. Transformational change could occur at the personal or individual level, but also at a wider, societal level ‐ among families, social networks and communities.

***Personal transformation**:* The personal transformation was characterised by changes in self‐perception and self‐identity. Interactions between varied global majority groups fostered friendships and opportunities for intercultural exchange, such as language learning and exploring different cultural traditions. Participants' increased confidence encouraged them to try new creative activities. One person, who joined a knitting group through a friend, later explored singing and drama—showing how success in one activity can act as a springboard, creating a cascade effect of wider engagement.

Participants began to experiment with new identities by challenging limiting personal beliefs. For some this meant seeing themselves as a creative person or artist rather than simply ‘someone who does art’. They began to surface and discuss experiences of discrimination, domestic violence and exclusion through their artwork, and to challenge ideas about ageing; this could engender a healing process as emotions were discussed within a safe environment. These vulnerable moments required skilled workers and volunteers, who noted the challenge of balancing activity delivery with the time and sensitivity needed to support individuals during and after sessions.

***Societal transformation:*** The impact of cultural participation extended beyond the individual. Family members expressed pride and saw their older relatives in a new light. Concerns about isolation or lack of purpose were often replaced by recognition of their relatives' skills, relationships, and meaningful involvement.

Beyond the family context, there were examples of performances that addressed issues relevant to ageing, ethnicity or gender norms, challenging stereotypical narratives of decline and passivity in older generations. Taking a role in spaces previously thought to be the preserve of the white British population highlighted older adults from global majority groups as dynamic contributors to cultural and social life. Participants were proud of the positive community response to their creative advocacy. Respectful attention to faith‐based norms allowed performance work to be adjusted sensitively and appropriately (see Table 9 for CMOCs relevant to Pillar 3).

### 3.6 Final Programme Theory for TOUS

**Figure 1:**
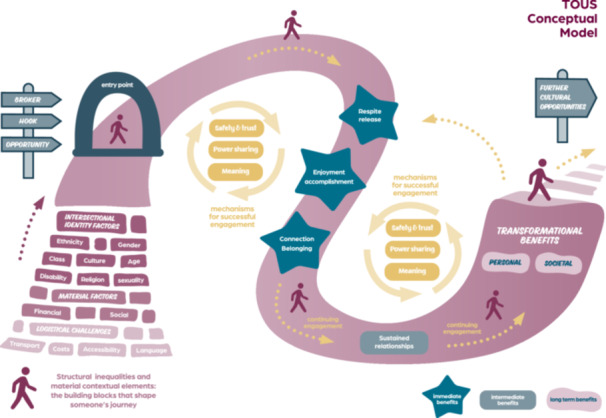
The three pillars of the TOUS programme.

The three pillars of the TOUS programme theory are shown in Figure 1. The model is represented by a cultural journey which an individual embarks on. They may need support and encouragement to enter the cultural space (Pillar 1). Then, the right environment is required for them to continue participating—provided by other participants, by the artists and facilitators, as well as aspects of the place (Pillar 2). Through engaging, a range of benefits might ensue over time (Pillar 3). This model does not presume that all individuals proceed through all stages (immediate, intermediate and longer‐term benefits); some may be content experiencing immediate benefits (e.g., respite, enjoyment). Nor does it assume that individuals go through the journey in a linear fashion or at the same rate but shows the possible trajectory. The journey may continue with people seeking other cultural opportunities or they may stay with the offer they embarked on.

This programme theory is informed by an ‘enabling places’ theoretical framework [35]. This framework considers the qualities of place that facilitate health and well‐being. It draws on three forms of resources—social (e.g., networks/interactions with others), affective (e.g., a sense of safety and belonging), material (e.g., artefacts such as art materials, food and drink, the physical environment). A sense of hope is stimulated through encountering pleasure and accomplishment alongside others. In the TOUS study, these resources were brought together by artists, facilitators, volunteers, and participants through a collaborative process.

## 4 Discussion

Studies show that cultural activities can benefit older adults' health and well‐being [1], but global majority groups remain under‐represented [8, 36, 37]. Implicit assumptions tend to reflect white, middle‐class norms and fail to resonate with the experiences, cultural traditions, diverse languages and expectations of global majority older adults. This has implications for link workers, who might consider connecting people they support to cultural spaces as part of a social prescription.

The TOUS study examined how cultural providers can better engage older adults from global majority communities by making offers more accessible, acceptable, and appropriate. Our research identified important and practical ways employed by participants themselves and organisations to enhance engagement. Participants described meaningful experiences with culturally relevant programming, especially when activities were co‐designed, delivered in familiar and/or welcoming spaces, and facilitated by trusted and culturally sensitive individuals.

### 4.1 Comparison With the Existing Literature

Our findings suggest that older individuals from global majority backgrounds engage with cultural activities through a two‐step process, similar to the stages described in other studies of equitable access to culture as: the ‘decision to go’ and the ‘decision to stay’ [3]. Our data highlighted that the decision to go depends on mobilising both personal and community resources and aligning three key elements: the broker, the hook, and the opportunity. The decision to stay rests on ‘enabling places’ [35] within the provider organisation – based on the capacity to build a sense of safety and trust, to foster power‐sharing, and identify meaning and purpose for participants in what is provided.

The benefits of engagement with cultural activities described in this study align closely with frameworks of psychological resilience [38], as well as more general health and well‐being outcomes cited in existing research [1, 29]. An arts‐through‐social‐prescribing programme found benefits for adults diagnosed with mental health problems in building social connections, respite from the everyday and enjoyment in playfulness [39]. Likewise, a heritage project found volunteer benefits included sense of purpose, self‐nurture and sharing with community members [40]. Although existing studies are mostly small in scale, there is growing evidence of the therapeutic benefits of activities in the cultural sector [41, 42, 43].

‘Threshold moments’ or turning points, where something shifts for a person in culture sector engagement, allow for the possibility of personal growth and change [39]. Improvements in self‐esteem, feelings of safety and empowerment have been found to lead to a ‘stepping stone’ to other activities for people [41]. However, while existing evidence shows that arts and culture can positively impact older adults' health and well‐being [1, 5], we found no other research specifically exploring how cultural activities that involve sharing and validating experiences of discrimination and exclusion can support healing in older adults from the global majority.

The possibility that cultural participation can increase social inclusion and social capital has been suggested [44, 45, 46], particularly in the international context [1]. TOUS study participants acquired social capital through the quality and structure of relationships and networks, which became a source of mutual benefit through trust, reciprocity, and collaboration [47]. This was particularly true for participants who were newcomers to the UK without English language skills or knowledge of societal norms. Feeling they belonged within a community that could support them in understanding the host society was critical. Both bonding social ties—which united culturally similar groups—and bridging ties [47] that connected more diverse groups with different traditions, were a key feature of the organisations serving migrant groups.

Beyond benefits to the individual, when older people were seen participating in public‐facing cultural activities, such as performance, they challenged prevailing societal assumptions about ageing, capability, and relevance. This disrupted stereotypical narratives of decline or passivity, highlighting older adults as active contributors to cultural and social life [48]. Their visible participation affirms that meaning, creativity, and personal growth are not bound by age [49]. In doing so, older people not only redefine their own identities but also prompt wider society to reconsider their value and potential as meaningful participants in communal and cultural spaces. As far as we could determine, the disruption of assumptions about the intersections of older age with other aspects of identity through cultural activity remains under‐explored in the existing literature.

### 4.2 Implications for Practice

Meaningful engagement in cultural activities for older people from global majority backgrounds, with its corresponding benefits, requires sustained, culturally sensitive work from providers and institutions to create ‘enabling places’ [35]. Barriers to cultural engagement reflect the complexity of individuals' identities and lived realities. Some organisations in the TOUS study addressed this by working with one specific ethnic or language group at a time; others took a broader approach, fostering connection through group activities, family engagement, and shared cultural events like festivals. If the mechanisms we identified were present (safety and trust, power‐sharing, meaning), both approaches could be successful.

The underpinning of relationship‐building, both with individuals and communities, alongside understanding of, and sensitivity to, cultural traditions and reference points are paramount. Training is needed to support trauma‐informed practices and enact safeguarding and anti‐discrimination policies. However, this work is time and resource intensive and requires adequate and sustained funding. We have shown that in order to accrue deeper and more transformational benefits, sustained relationships and repeated engagement is required. It is demanding on personal resources and skills of workers, volunteers and artists who hold these safe spaces for people who may have multiple talents and strengths, as well as diverse vulnerabilities or needs. It has been argued that the moral weight of responsibility for holding the emotional response of individuals, as well as supporting their physical and access needs, is disproportionately laid on the shoulders of artists and facilitators and is a largely hidden cost [50]. Organisations seeking to follow the examples of good practice need to consider carefully how this might impact on their capacity, and funding bodies need to consider the implications of short‐term funding.

Intermediaries such as social prescribing link workers could play a key role in facilitating access to cultural sector activities for older people from global majority groups. However, there were few examples of referrals by link workers to the cultural spaces involved in the TOUS study, and brokerage was mostly from within people's own communities. This may be because link workers are not aware of the benefits of cultural activities, or they may unconsciously filter opportunities based on assumptions about individuals' interests [21]. This highlights the need for reflexivity and training within referral pathways to ensure inclusive and equitable social prescribing.

The TOUS programme theory serves as a prototype for groups and organisations, offering guidance on key factors to increase inclusive practice. It can support programme planning, funding applications, ongoing evaluation, and alignment among those delivering activities [51]. The next phase of our research will be developing an online implementation tool, based around the programme theory. In addition, further research is needed to establish whether this programme theory might be applicable to people with different characteristics who may feel underserved by cultural spaces (e.g., people with a learning disability or visual impairment).

### 4.3 Limitations

This study's geographic scope and sample size may limit the transferability of our programme theory. Despite employing a range of recruitment strategies, we were unsuccessful in identifying research case sites in Scotland and Northern Ireland, but key informant interviews were carried out with individuals from these locations. Future research should further develop and test the programme theory drawing on data from diverse contexts and cultural groups. Longitudinal studies could provide insight into the sustained impact of cultural engagement on well‐being in later life.

## 5 Conclusion

While there are clear barriers to engagement in cultural activities, groups and spaces for older people from global majority backgrounds, this research demonstrates that with intentional engagement and power‐sharing, organisations can foster participation and support well‐being in later life. Our programme theory begins with the overlapping principles of the broker, hook and opportunity (Pillar 1), necessary for stepping over the threshold into a new cultural activity. The intention of people to stay engaged, once crossing the threshold, is secured by cultural organisations themselves: through the mechanisms of safety and trust, power‐sharing and meaning (Pillar 2). The theory shows that beneficial outcomes (Pillar 3) may be immediate, intermediate, or long‐term, depending on an individual's engagement with activities and the people organising and attending them, and will vary from person to person. Relational work underpins all pillars of the programme theory, and our examples show that with adequate resources, community‐led or partnership‐based approaches can foster more inclusive and responsive cultural offers.

## Author Contributions

**Debra Westlake:** formal analysis (equal), investigation (equal), writing draft (lead), editing and reviewing (lead). **Kamal R. Mahtani:** conceptualisation (equal), formal analysis (equal), methodology (equal), editing and reviewing (equal), financial acquisition (equal). **Geoffrey Wong:** conceptualisation (equal), formal analysis (equal), methodology (equal), editing and reviewing (equal). **Jordan Gorenberg:** formal analysis (equal), editing and reviewing (equal). **Marta Santillo:** formal analysis (equal), editing and reviewing (equal). **Kerryn Husk:** conceptualisation (equal), formal analysis (equal), methodology (equal), editing and reviewing (equal). **Sofia Vougioukalou:** conceptualisation (equal), formal analysis (equal), methodology (equal), editing and reviewing (equal). **Ruthanne Baxter:** conceptualisation (equal), formal analysis (equal), methodology (equal), editing and reviewing (equal). **Shoba Dawson:** conceptualisation (equal), formal analysis (equal), methodology (equal), editing and reviewing (equal). **Caroline M. Potter:** conceptualisation (equal), formal analysis (equal), methodology (equal), editing and reviewing (equal). **Harriet Warburton:** conceptualisation (equal), formal analysis (equal), methodology (equal), editing and reviewing (equal). **Beth McDougall:** conceptualisation (equal), formal analysis (equal), methodology (equal), editing and reviewing (equal). **Stephanie Tierney:** conceptualisation (equal), investigation (equal), formal analysis (equal), methodology (equal), editing and reviewing (equal), financial acquisition (equal).

## Disclosure

The views expressed are those of the authors and not necessarily those of the funder or the authors' host institutions.

## Ethics Statement

Approval was granted by the University of Oxford's Central University Research Ethics Committee (ref: R90223/RE001).

## Consent

Verbal consent was obtained from interviewees.

## Conflicts of Interest

The authors declare no conflicts of interest.

## Supporting information

TOUS_ Supporting references for CMOCs.

## Data Availability

The datasets generated and/or analysed during the current study are not publicly available due to confidentiality and participant privacy, but summary data are available from the corresponding author upon request.
