# Supplementary material for: Delivering Inclusive Cultural Offers for Social Prescribing: A Realist Evaluation Involving Older People From Global Majority Backgrounds and Cultural Sector Providers in the UK
Source: Health Expect. 2026 Feb 14;29(1):e70550. doi: 10.1111/hex.70550 (PMC12906276; doi:10.1111/hex.70550)
Supplement: Supplementary file 1 — TOUS_ Supporting references for CMOCs. [file HEX-29-e70550-s001.docx]

***PILLAR ONE***

***Supporting references for CMOCs on ‘broker’***

Site 1 P07 story: “I’m walking up the high street, if I see someone I know and I try to tell them they say oh, like they don’t think they could be bothered with coming. I tell a couple of people and they come and then they won't come back again, I don’t know why.”

Site 3 P04: “I started doing some outreach service at a care home, an elderly care home, and that does have, you know, doing like a poetry reading session, and they do have Black and Asian people there, you know, residents.”

Site 4 P02: “…we find that it works really well; people who are known and trusted within their communities, who then go and talk to people that they know, and spread awareness of these things.”

Site 5 P02: “I was a bit apprehensive as anybody is going into a new place and meeting new people but I think after, I just walked into the reception, saw [name] and we bonded immediately and she took me downstairs, we talked with everybody…”

Site 6 P02 story: “My daughter gets some information about the [theatre], that the Elders Company wants to get some new members. She said, ‘Pap, go to try!’”

Site 6 P06 story: “I heard about the [theatre group] as I'm in a few women's groups on WhatsApp…it was a workshop for four weeks at the library. There's a cafe there, which is where I met [producer] and the other team. They said we're going to make a film, and I thought, oh this is exciting!”

Key informant interview P09: “…what we did was what we tend to do if we’re going to try and reach a new group is work with the Open Museums. The Open Museum can go out, do outreach sessions with them in their own environment, which I think is really important for your first session that you're going out into their space in their community, and kind of building those bridges before you try and bring them in. So, like what could be like an alien like environment coming to museum they've never been to before.”

Site 1 P08 (story): “I’m very positive, so wherever I go I like to make an impact. I like people, I like socialising, I like making people feel valued, and I like being friendly. So my positivity carries me afar. Because it’s in you – you have that confidence, you are proud of yourself, you are proud of what you do, you are happy within . That happiness, you bring it out, you impact it on people.”

***Supporting references for CMOCs on ‘hook’***

Site 1 P06: “…one of our long-term partners has a real focus on West African drumming and dance movement, and that attracted new members to the group from those backgrounds. Then we have our choir facilitator, who brings people's cultural backgrounds into the sessions, which I think’s really key for people to feel heard and listened to.”

Site 2 P02: “…culturally, we come from the same places, so we know what's, you know, the language is the same, so there’s not too much explanation that has to be done. They can use things that are familiar, you know, foods and colours and situations that would be familiar to them in their own countries, in their growing up, you know, plants, and flowers, and herbs

Site 5 P06: “I’m from the Bangladesh community and I can see a Bengali mother walk into a library whose English is second language and maybe cannot read or write, it could be daunting. But I think if you have a staff who looks like them it makes life much more easier, and I think our staff force is beginning to reflect that.”

Site 6 P09 story: “If you want to attract a Muslim community if you have something religious, then that would really make them interested. Also, if you want to bring them in, just give them something according to their interest…”

***Supporting references for CMOCs on ‘opportunity’***

Site 1 P03 story: “After the pandemic I was keen to get back out into the world, meet new people and develop my confidence.”

Site 1 P07 story: “I would get off at the front on the bus and I walked down and then looked and I always see people standing outside at the building. There is a lady I know, and I said to her, “what’s going on?” She told me that they have a club… [I thought] “that’s a good idea, when I’ve stopped work, I will try it”. And that’s what I did. I put my name down.”

Site 5 P02: “…sadly her father-in-law passed away last year and I think that gave her more time and now she comes in for other things.”

Site 5 P02: “…during COVID that everybody was on lockdown and I normally have my grandchildren every weekend and all the holidays...So I was sitting here and I was feeling really depressed and out of the blue somebody that I’d known for years phoned me up…I said to her that I was…a bit depressed and very lonely. So she said there’s a group called [organisation]…she said well during COVID they’re doing like yoga classes on Zoom and IT classes and I thought oh right, okay, that will stop me from sort of turning on YouTube and doing it on my own.”

Site 6 P03 story: “I worked for the NHS, but I was totally drained so I took early retirement. Then I realised I've got to give something back to myself. I’ve got to embrace whatever creativity is buried in there, it's got to come out.”

Site 6 P06 story: “I've always been interested in arts and culture, but I never tapped into it. There was one time, when the library was still on the main road, that I met this elderly gentleman who was painting with water colours. He had his table, his paints, and was just painting. I thought, am I in the right place? This is the library. But he said to come over and started teaching me. I did two canvases, and he said I was really good at this.”

Site 6 P10 story: “All our other projects for older people, there's a fee to take part. For the Elders’ projects we were really clear that there wouldn't be that barrier.”

***PILLAR TWO***

***Supporting references for CMOCs on ‘safety and trust’***

Site 5 P01: “100% the space that you do it in is very important and the environment, definitely, the women feel very safe there. A lot of them will take hijabs off because they feel comfortable to as well, so they can be themselves.”

Site 4 P04: “Everybody at the museum is so welcoming, there's nobody that I don't think has ever turned around to me anyway that have said, ‘Well, I don't feel welcome when I come into these doors,’ and it is having that great team behind you as well, or with you, it’s having that welcoming team within that space as well.”

Site 2 P02: “I think first of all, you know, to create a safe space, you need to feel safe. And one of the ways that you can feel safe is if you trust, you know, the facilitator, trust not just the facilitator, but trust the people around you as well, getting to know each other. That's the beginning. I think that's the initial, that's I think where you would start. So, there have to be some kind of consistency in, you know, if you're meeting every week and you're seeing the same people every week, you're getting familiar, and then you start to feel comfortable, get to know each other.

Site 1 P05 “I’ve visited other centres, and I literally feel at home here. I had an issue where I didn’t really trust anyone and therefore, I would shut myself off and not talk to anyone, but that’s gone now.”

Site 6 P03 (story): “Initially I was very nervous. When I'm in new surroundings and I don't know anybody the way I keep safe is by listening. I don’t say anything. But then our director, who is absolutely wonderful, read out this safe space statement. I could feel my body relaxing and I thought yes, this feels okay. We talked about the Elders Company, and I knew I’d really love to be involved.”

***Supporting references for CMOCs on ‘power sharing’***

Site 6 P05 (story): “But it's also taught me how to let everyone have their voice. You need to be fair and yes, sometimes we need to compromise but everyone has a chance to say what’s on their mind. If you look at the drama people will choose who the main character is. But everyone is the main character, everyone backstage too. The whole show is their creation, the writing, the painting, everything.”

Site 4 P01: “what we hadn’t realised before we set out on this Mondays at the Museum [museum activity] was that there’s actually quite a high Afro-Caribbean population in that area across the road, but they had never ever engaged with the museum. One of the guys that comes regularly now I’d spoken to and he said I’ve lived across the road for 50 years and never been in the museum! [laughs] To me that is one of the really important markers of success, because they’ve never felt welcome, it’s never felt like a place for them before. But now we’re offering them something that they want, they’re able to come and make it their home if you like, as well.”

Site 3 P05: “I don’t think we think in terms of age groups generally, we don’t think in that way. We listen to the community and we think, there’s a need there, and then we act. We don’t think, is this going to be for an older person or a younger person or a- I mean, obviously we have a children’s offer and we have an adults’ offer, but we don’t necessarily fine tune it down to, you know, will this attract an older person or won’t it.”

Site 2 P01: “It’s the sense of empowerment, a sense of taking nothing and turning nothing into something that's tangible and beautiful. For real. For real. For real, because if I showed you and I placed stuff on the table that, I mean tomorrow, I've got an idea of where I want to work with cardboard, but I want to create a piece of sculpture. But I also want to use the cardboard to create chains, rusty chains that will make the hair. Now, if I put the cardboard down and say that we're going to do, most people think we're mad. But in that sense of achievement, it's that sense of achievement. You know, from nothing to something.”

Site 2 P02: “Yes, well, we do, we do consult, you know, we have members meetings with our members where we do ask them, you know, to come forward with their ideas, what they would like to do. You know, so some of it I would say, you know, has been as a result consulting with them. And them probably, you know, maybe experiencing similar things at other clubs. Yes, for some of them, you know. And yes, just wanting to try something different, you know.”

Site 1 P01 (story): “I always tell them it's not good for you sitting at home, doing nothing or watching telly. You come out and help yourself. You are helping yourself. It's not everything the doctor will, the doctor can only prescribe for you or give you medicine, or tell you oh, you should have that 30 minutes walk, oh, you should do this. No. You yourself have to help yourself. When you tell people about what you are doing and all that, that makes them to get the mind of leaving home, of helping themselves as well.”

***Supporting references for CMOCs on ‘meaning’***

Site 4 P05: “the activities have just been amazing because it’s good for your mental health, creative arts, and the drumming, everything. It’s a bit of everything for everybody. So we used to get people that used to just come for one activity in the morning, but now they’re actually spending most of the day and they bring their lunch with them as well, so it just talks for itself.”

Site 6 P01 (story): “Oh, I have rushed to get to Elders at the Royal Exchange[theatre] because I am telling you I was empowered! It doesn’t matter how tired I was when you get here, you are in stitches with laughing. There’s always something funny to make you laugh. It was a pleasure becoming an Elder so many excitements!”

Site 3 P01: “But it's amazing from going to see that because the museum was taken over by a black cultural organisation, and then people asking questions and people saying, well, you know, I came here as a child, but I didn't feel that I belonged here. But now, I'm coming here and with more people of colour within that environment, all appreciating the world, I feel that I belong now.”

Site 1 P05: “It makes me feel like my day has been fruitful and when I go home I don’t have this thought of reflecting back on the past negative experiences. I feel like what we do here, it gives me a happy feeling.”

Site 1 P01 (story): “People love it and when they were clapping [laughs]. I feel good . Yeah. I feel good, you know... They [my family] are proud of me. I feel happy that I'm doing something that they like. So, when I'm coming here doing acting play and all that, I'm so happy. And I’ve got the interest... Because if you don't love something, you won't do it. It's true love when you do things, if I come here and I don't like it, I won't come back.”

Site 1 P08 (story): “Doing this kind of thing is important because it eradicates loneliness. Because you go out and you meet people. You socialise, you get involved, and by the time you get home you are tired, you are exhausted, and you’ve had a good day. The days just feel like that. It’s better than sitting all day watching television, doing that and getting bored.”

Site 6 P01 (story): “It doesn’t matter how tired I was when you get here, you are in stitches with laughing. There’s always something funny to make you laugh. It was a pleasure becoming an Elder so many excitements! A self-belonging without knowing it.”

Site 1 P05 (story): “When I wake up on a Tuesday morning my first thought is on Meet Me at the Albany[theatre activity] and it’s a motivation knowing that it’s something to look forward to. I’ve visited other centres, and I literally feel at home here . I had an issue where I didn’t really trust anyone and therefore, I would shut myself off and not talk to anyone, but that’s gone now.”

Site 1 P04: “I look forward to coming here because you learn so much from the people, it’s such a lovely feeling. It’s good to be able to express myself in such a fashion now compared to before. I used to sit there and think I can't do this, and I don’t want to do that – but now I can't stop! Everything works out in the end, because of where I am today.”

Site 1 P01 (story): “We look forward to Monday, we look forward to Tuesday. It is a part of our body that we look forward to. Where we come, we see our age group . We sit down, we chat, we drink tea, we eat biscuit, you know, we chat, before we start, you know, everything. That elevates your spirit. It elevates your mind. You know, it makes you feel happy that you’ve come to join your age group. And you are working together as group.”

**PILLAR 3 - BENEFITS**

***Supporting references for CMOCs on Immediate benefits***

This was at a point in my life where I was going through quite a lot, and I knew I needed a distraction. (Site06_06)

It makes you smile, makes you think, makes you feel more patience. I mean, this is what happens to me. A relief from everyday work. (Site05_08)

It’s a chance to get out of your own headspace. You don’t talk about individual problems; you just focus on the creative activity. (Site01_09)

When you focus your attention on some subject, you can forget everything. Make your mind calm and clear. This is in Chinese called ‘Zen.’ Another factor, because of the painting, you keep your hand and your brain active, the nerves. Another factor is about creativity. If you make something new, this allows your mind to expand. It makes your brain more active and alert.

(Site06_02).

Well, I put it, it's like love. It's love because when you going somewhere, and you love what you are seeing or what you are doing there, I put it, love. It’s a sign of love (Site01_01)

It’s good to get together with ladies of your own culture, because nowadays we don’t get enough time for ourselves. It can get so busy in our own household stuff. We need a couple of hours, just for us (Site06_07)

It makes me feel like my day has been fruitful and when I go home, I don’t have this thought of reflecting back on the past negative experiences. (Site01_05)

When I go back home, I feel more like I'm me. I am happy that I did something during the day, you know, with others. Out of the house, getting dressed, putting a little bit of makeup. All these tiny things make a difference, you know? (Site05_08)

We’re just all ladies together, I think that inhibitions have gone, you see the ladies that wear the niqab and the long hijab and that’s all, headscarves are off, everything is off, the door is locked, and they’re in t-shirts and jogging bottoms and they’re going for it, you know?[…] So no one can criticise, everyone is the same, no one looks at you thinking, oh my god, look at her, […] you’re in a safe space, just let go and if you don’t know the words, make your own words up like I do. You know, it’s just letting them, all their inhibitions gone, being free and having that time for yourself. (Site05_02)

In the Muslim religion dressed like I am […] I’m in shorts and t-shirt, but in my jogging bottoms, this is what I wear at home, short sleeved t-shirt and my jogging bottoms, and I can open, because it’s not low-cut, it’s not showing, only my arms, I can open the door to any Tom, Dick and Harry that walks in through my house but if I know that I’m expecting another Muslim person I will wear longer sleeves. I know my sisters wear the headscarf, I never have so I don’t have to worry about before I open the door put my headscarf on, make sure that I’m not showing any skin, you know, make sure that the headscarf is covering any skin that’s showing, but this is what the ladies have to do in their homes. So being in an environment like that where you can be totally free knowing that that door is shut, no one is coming in, is amazing.(Site05_02)

The sessions gave the group space for self-reflection. It's just like drama therapy and I'm a counsellor, so I think it's so powerful and exciting. For example, when the group was creating the story they straight away came up with what they wanted to say. Maybe this had been something in their head for a long time, but they had no way to express it. (Site06_05)

But making the film triggered so many hidden emotions. My mum had passed away ten years ago, but I’d never really grieved properly, because when my mum sadly passed away, my younger sister became very ill a couple of weeks later. She had cancer and we were supporting her. I didn’t really acknowledge my feelings until I did the workshop. I was honestly in shock at how much I’d buried inside of me.(Site06_06)

And it’s sad that there is things that lie dormant in me and it was like when I came back here it’s like, it’s awoken. (Site01_05)

But A and his team were amazing in helping me with that, signposting support, and I felt as if I was in a family – we all looked out for each other. […] I want to look after me and enjoy all the blessings that Allah’s sent me, because we don't know if we're going to wake up in the morning. I was in such a dark place, but it was like a light had switched on in that workshop, and I never really looked back. It came at the right time. .(Site06_06)

It's kind of a catharsis for them, verification of their thoughts or feelings. Because when they give an outlet of their emotions and feelings, they feel like they are something; that’s why they are asked to tell their story so they feel now they are being involved in something as a part of a whole. So it gives them motivation. (Site06_09)

I mostly like doing the art side, I like the dance group, anything that’s to do with movement, anything that’s motivating or keeping your mind active because a lot of the things we do here is keeping your mind active. It’s just when you’re doing the things, you’re doing it in a relaxed kind of way, somehow, it’s like everybody is doing the same thing and everybody is connected, and you can see everybody is enjoying what they’re doing. (Site01_05)

It’s good to get together with ladies of your own culture, because nowadays we don’t get enough time for ourselves. It can get so busy in our own household stuff. We need a couple of hours, just for us. (Site06_07)

I was so happy people speak the same language, and you learn from different accents and from different people. There is prejudice everywhere in the world, regardless of whether you’re black or white or whatever, but I didn’t find that here. We’re like one body together. (Site01_04)

This is where my creativity is. This is home. I belong here. (Site06_03)

All the women there make you feel like a second family. Everyone is equal and welcoming because we all from the different countries, and our language level all different. We are patient and try to help each other so we don't have any fear of discrimination. (Site05_04)

The stories describe experiences of isolation, loneliness, and social disconnection, exacerbated by the covid-19 pandemic but also resulting from other factors including declining health and mobility, lack of services and community support, lack of access to transport, living situations, and bereavement. For many of the storytellers, racism and gendered discrimination had also limited opportunities both in the past and the present and contributed to a sense of exclusion. (extract from OFS Storytelling report from Site01, p. 7)

I’ve seen her life flourish. I think she just felt we all so supportive, we don’t see her as she's got a problem, but we see her as part of the group. Whatever her past, we don’t look at that. We know everyone is trying to get some sort of support. And because I was on the group, I felt by talking to each person I now realise that everyone has something that really does affect them in their daily life. (Site03_P02/P03)

***Supporting references for CMOCs on Intermediate benefits***

Coming to (Site01) has been a blessing for me, it’s made me feel that I can do things, I could explore my mind and continue to do things at home. (Site01-06)

For many members, this means that personal talents and ambitions, often suppressed or neglected in the past, can be revisited, leading to feelings of great satisfaction and fulfilment. Storytellers describe experiences of transformation and self-discovery and renewed confidence as a result of taking part. (Extract from Storytelling report Site01 p17)

I feel now I want to be out of the house. I used to be at home for my children, for the house, for my husband. Now it is my time. I think now it is my time to do something, to see where I am. There is something inside me – I want to build my confidence (Site05_08)

When you’re talking to other people you forget about your own stuff. You hear somebody else’s problems then you’re thinking oh no mine’s nothing in comparison. It’s a good thing to share. When you’re talking to someone that you can trust, you can let your hair down a little bit, and that takes a little burden off your chest. (Site06_07)

We know how to treat each other. The relationship between us, it's not a client or a participant. [..] It’s connecting. It’s really building that relationship. These women are so precious. We've all left our countries at a certain age. We're all here without our families. [...] It's their experience that gives me support. We became like a community ourselves. I feel these women are my family. (Site05_05)

But as I come here sitting with people, we chat, we recognise one another, we exchange telephone number, you know, phoning them. Like if they don't come, you phone them. What happened? You didn't come to ((organisation))? What happened? Oh, I got doctor's appointment or got hospital appointment or, you know, that is why. Okay, you're okay though? Yeah. Good. You know, as I said, it's love. You know. (Site01_01)

Storytellers describe sessions as welcoming, inclusive, and friendly. They use warm, emotive words like ‘family’, ‘love’, ‘blessing’, ‘camaraderie’, ‘heart’, and ‘soul’ when talking about how attending makes them feel. Reasons for this include an accessible community space, kind and attentive staff and volunteers, a holistic approach in which social, physical, and intellectual needs are considered, a culture of listening and co-production, and an ability to be flexible and make adaptations to meet individual need. Members have also created a network of peer support, encouraging each other to attend and participate and checking in between sessions. (Extract from Storytelling report Site01 p11)

I look forward to coming here because you learn so much from the people, it’s such a lovely feeling. It’s good to be able to express myself in such a fashion now compared to before. I used to sit there and think I can't do this, and I don’t want to do that – but now I can't stop! Everything works out in the end, because of where I am today. (Site01_04)

When I wake up on a Tuesday morning my first thought is on Meet Me at the Albany and it’s a motivation knowing that it’s something to look forward to. I’ve visited other centres, and I literally feel at home here. I had an issue where I didn’t really trust anyone and therefore, I would shut myself off and not talk to anyone, but that’s gone now.(Site01_05)

I think the biggest thing is confidence building. To have, yes to feel confident to express, to be sociable. I think also, I think people gain a sense of motivation and purpose, and sometimes that then translates into actually now I wanna go and do something else, this other thing or actually yes, I've outgrown this like, so I think, confidence, motivation. (Site06_10)

The impact is very positive for me, through the activities here. I’m more confident to do things, more confident to explore opportunity, to take part in projects. (Site06_02)

When I go back home, I feel more like I'm me. I am happy that I did something during the day, you know, with others. Out of the house, getting dressed, putting a little bit of makeup. All these tiny things make a difference, you know? Gives you more energy, to be more happier. Because if you just go buy clothes for the wardrobe... No, you want to show your friends. You need to be out having coffee. Something different, yes. It also affects the health. At home you feel you are doing like just washing, cleaning. But when you come here, having a chat, having a laugh makes a lot of difference, you know, to your feelings. (Site05_08)

It’s not the same each week, which sometimes is confusing, but largely this is a safe space to try different things. Variety is important because it helps undo the stereotype of older people being resistant to change. There is something about challenging people, like very recently with the lino printing – people are surprised that they've done it. It gives such a sense of achievement. They’ve created something new and they’ve never done this before. (Site01_06)

I never know how to draw, never in my life. But they have some pictures that I’ve drawn. I can't believe it’s me who did that drawing. I also did a lot of knitting, crocheting – I can do all sorts. [...] There are so many things to do, that if I don’t feel like doing drawing, I’ll do something else. I don’t think anything can be improved about the programme. Somebody is always willing to help you. (Site01_04)

The third week of the choir, the man who organised the drama came to me. And he said, we have a drama here on Monday. Are you interested? So, I said, well, I don't know if I'm interested or not, but I will come and see. If I like it, I will stay. If I don't, I don't have to come. He said that's fine. And I went from that day till today. I never looked back. So, every Monday and Tuesday, I'm looking forward to come here. I’ve never done anything like drama before. (Site01_01)

***Supporting references for CMOCs on longer-term transformational benefits***

The creative process is very healing. I used to work predominantly with women, men and children that have been raped or sexually assaulted. I worked in Sierra Leone after the Blood Diamond War had ended, and in Uganda with child soldiers. When I came back, I was totally desensitised. I felt nothing and it was scary. I worked for the NHS, but I was totally drained so I took early retirement. Then I realised I've got to give something back to myself. I’ve got to embrace whatever creativity is buried in there, it's got to come out. Being here is like nurturing myself. It's a lovely, lovely feeling. (Site06_03)

When I came here, I feel privileged to come. But when I see the way people are behaving, the way we’re treating ourselves because of colour is ridiculous. It's only the skin. The blood is red, like your blood. It's just the skin. But they don't see us as them. My husband won’t let me go back. He said you just need to ignore them, and that's what I did. It was difficult, but we survive it and God put us through. That's why everything I do, I give glory to God. Gradually things are improving but not yet. There’s still a long way to go[…] I didn’t find any racism here to be honest. No. It might be, but I didn’t find it. (Site01_ 02)

Just because our South Asian community is a minority they should not be neglected. So, if we ask them to tell their views, to tell how they feel, they will feel like more inclusive environment around the community. I think because our culture is totally different and they want to keep their roots, they want to keep their culture whatever it is. Some of the modern generations they're trying to mix but still our parents they want the same culture growing up. (Site06_09)

If I want to introduce someone to the group, I will tell them it’s a totally different small world. You need to come and experience and here you can make new friends. You will know the different history and traditions, and then you can improve your English as well. [...] so that's all benefit for your personal development. (Site05-04)

Such positive impacts [from being involved at the organisation) include improved mental and physical health, increased confidence, a sense of time and regularity, a feeling of belonging, opportunities for personal growth and learning, (Extract from Storytelling report, Site01, p9)

There is something inside me – I want to build my confidence. Even my husband said ‘you are too shy – too shy to say something, like if anyone did something wrong to you, you don't answer back.’ I know. I know. But I learnt here – you have to speak out. (Site05_08)

I want to make my mark in the world through my passion for creativity, for theatre. I don't feel afraid anymore or that this isn't for me because of my colour or my class. I feel unstoppable. (Site06_03)

when the group was creating the story they straight away came up with what they wanted to say. Maybe this had been something in their head for a long time, but they had no way to express it. Through this training, they realised their talent. I learned so much from this process. I learned a lot about drama and the process of developing a story. But it's also taught me how to let everyone have their voice (Site06_05)

When I came here in the 60s, I wanted to act. I applied to Hollywood, and they asked me to come down. They tell me when to come and how I should prepare myself, that they are going to take pictures. So, I went to the hairdresser, did my hairdressing very well, put on nice clothes, and I went. When I got home, they wrote a letter to say, congratulations you are chosen to come down, whatever you want to do, either singing or acting play. I told my husband. My husband says, No way, you're not going. I begged, and begged, and begged. He refused. I was really angry, to be fair. I was really, really angry because that's what I wanted to do in life. And then he refuses. So, when I'm here acting my son says, Mom, this is what you wanted to do but Daddy didn’t let you do it. Now, you are doing it, and I’m proud of you. (Site01_01)

Such positive impacts [from being involved at the organisation) include […] the rediscovery of interests or talents that had been suppressed or neglected (Extract from Storytelling report, Site01, p9)

I have now learned a lot and able to share this in the community. I have come to shows and reading and understanding more about the shows. I am having a better cultural awareness that as a Black woman I can do this. There is no stopping you. (Site06_01)

I had felt rubbish because when you join a society people ignore you. They think you are not good, no skills to communicate. You feel like, oh I'm useless. But since join this group I never have this feeling. As part of this group, you have the opportunity to express yourself and show your skills. (Site05_04)

In my experience as a black woman many people don't give you the opportunity to shine. You absorb racism from when you’re little and you carry it because it's so damaging, it leaves scars. So, this for me was a two-way process. I was allowing myself to be creative in front of predominantly white people, even though I was scared of not being accepted or my creativity not being good enough. But then somebody sees something in me and thinks yes you do shine. Come and join the family. And here I am. (Site06_03)

For many members, this means that personal talents and ambitions, often suppressed or neglected in the past, can be revisited, leading to feelings of great satisfaction and fulfilment. Storytellers describe experiences of transformation and self-discovery and renewed confidence as a result of taking part. (Extract from Storytelling report Site01 p17)

There’s also this idea that older people are always looking back to the past. Which is the case sometimes, but there's a real curiosity in the members. If we’ve got new volunteers they ask, oh where are you from? What have you done? What do you do outside of this? And that confidence and curiosity comes from feeling safe. (Site01_06)

In another play, the council are forcing people to move from where they live. Strong hearted woman who live in a place for thirty years. And all of a sudden she have to move. Did she tell you she want to live closer to her daughter? You can't decide my life for me, you don't know my life, so you can't decide for me. I know my life. I will tell you this is who I am. Not you telling me. That was the message of that programme. This does happen to elderly people. (Site01_02)

I feel happy that I'm doing something, at my age I don't want to sit down without doing nothing because if you sit down doing nothing, the brain is sleepy. And that's why you see a lot of people having, what do you call it? The stress, and they don't know what to do with themselves. So, don't just sit idle because you are old. No, keep on doing something. That helps the body to be active and also helps the brain as well, right? You have to try and keep yourself busy. (Siter01_01)

When I joined the singing group I thoroughly started to enjoy it, and everybody enjoys it. I find the singing group, even if you can't sing, it makes you feel that you can sing even if you can't, and that’s therapy as well as for your lungs. I realised it’s to do with your emotions as well, it’s to do with your feelings. It’s not just about the singing it’s about the health effect on your body. (Site01_05)

And travelling from home to here, and here to home, actually, this is not a waste of time. This also make you feel better. When I come from home to the tram station it’s ten minutes of walking. And I said, ‘No, I want to run!’ I need to keep fit. I also do exercise in Tai Chi every day. Before, I’m a bit lazy to do that. But I want to keep my fitness for performing here. […] Also, physical wellbeing. Stronger, more flexible. Before, my lower back pain. But since I joined here, and I carry on with my Tai Chi and exercises, it’s gone. Before, maybe I meet the GP at least twice or three times per year. And now in just under two years I haven't been to visit the GP. […] Another factor, because of the painting, you keep your hand and your brain active, the nerves. (Site06_02)

I think the biggest thing is confidence building. To have, yes to feel confident to express, to be sociable. I think also, I think people gain a sense of motivation and purpose, and sometimes that then translates into actually now I wanna go and do something else, this other thing or actually yes, I've outgrown this like, so I think, confidence, motivation. (Site06_10)

The impact is very positive for me, through the activities here. I’m more confident to do things, more confident to explore opportunity, to take part in projects. (Site06_02)

I never put me in front, but that particular workshop made me wake up to the fact. I just wanted to give myself a chance, and from that couple of sessions, I started doing other things like going back into arts. (Site06_06)

***Supporting references for CMOCS on societal transformational benefits***

People love it and when they were clapping [laughs]. I feel good. Yeah. I feel good, you know. We're speaking up for elderly people, old people. Because everybody will get to that stage.

My feeling is that they should take care of the elderly people, they should look after them. And also, those that can come out, should be able to come out and not sit in at home doing nothing.(Site01_01)

When people laugh, it’s happiness. It’s happiness. Its de-stressing. It's very interesting […]You know, when you're doing something and people laugh, it's not a laughing as a laughingstock, it’s a sort of laughing that elevates them.(Site01_01)

In another play, the council are forcing people to move from where they live. Strong hearted woman who live in a place for thirty years. And all of a sudden she have to move. Did she tell you she want to live closer to her daughter? You can't decide my life for me, you don't know my life, so you can't decide for me. I know my life. I will tell you this is who I am. Not you telling me. That was the message of that programme. This does happen to elderly people. (Site01_02)

There’s also this idea that older people are always looking back to the past. Which is the case sometimes, but there's a real curiosity in the members. If we’ve got new volunteers they ask, oh where are you from? What have you done? What do you do outside of this? And that confidence and curiosity comes from feeling safe(site01_06)

We performed in the studio, and the audience liked it. They went, ‘Ah, amazing. This is good!’ Every time full, maybe about two hundred? My daughter and wife say, ‘Wow! Amazing! My dad is doing that.’ They were very excited. (Site06_02)

My family love it. My beginning was not as good as what I have now. They said after going through all that, God had blessed me with all this. So, yeah, they are happy. They always tell me they are proud of me. They always say, yeah, you are so popular now, we have to book appointment to see you. “Famous grandma”. (Site01-02)

And it's good, it doesn't let my children get worried. So, that why therefore, mom, did you go out today? I say, yeah. Where did you go? I went to ((organisation)). What did you people do? Then I will tell, this is what we did today. If we are acting a play, I say, oh, we are acting a new play. (Site01_01)

We’ve been working on it for a few weeks now. All my children they said to me it was good I was going out there and enjoying myself, because it’s not in our culture to go out there and present ourselves. (Site06_03)

But yes, I mean, you know, the positive effects that we have, you know, on our members and on their families as well because for most of them, for many of them, this is like a respite for (inaudible 41:56), you know, especially those living with dementia. You know, it's hard work. And so, to them, they're able to have their loved ones come and be away for, you know, the few hours a day, it's a big break for a lot of them, you know (Site02_02)

Then when you go back and express everything, you feel something different. Sometimes here we have like meeting special people, then I go back: ‘I met the Prime Minister of [country]. I did that. ‘Oh my God, mummy you are so famous now!’ My family are really, really happy for me. They say, ‘mum, you are doing good, go out. Be more active. Stop thinking about yourself, about the condition’(Site05_08)

Meanwhile to my family, all three hundred of them in [name of city]; I can say this age-friendly newspaper in Manchester, turn the page and you will see me there with a banner saying, “come and join the [name of theatre].” She is doing something in her old age. She can do it. (Site06_01)

And then thirdly, don't treat them like children. Really push the boat out, which is what I do with them as if they the students at the London College of Fashion or whatever, with art. Just completely just don't hold back. Don't because they're elderly or whatever, I don't do that at all. I don't believe in that at all. So, those are the kind of three things. And also don't, for me, with my group, and I can't speak for anyone else’s, I don't make it so it's overtly Caribbean, you know. Yes, we have a Caribbean flavour, and twinge in what we do, we push them further out to look at other things, to see other things.(Site02_01)

My relationship with ageing has changed a lot. My own consciousness around ageism has been raised. I've made work in new ways and about new stuff, that's non-text based and about people's lived experience.(Site06_10)
